# A case of long-term complete remission of locally advanced T4 bladder cancer treated with pembrolizumab

**DOI:** 10.1016/j.eucr.2021.101959

**Published:** 2021-11-27

**Authors:** Yuki Horibe, Wataru Nakata, Goh Tsujimura, Yuichi Tsujimoto, Takayoshi Gotoh, Masao Tsujihata

**Affiliations:** a Departments of Urology, Osaka Rosai Hospital, 1179-3 Nagasone, Kitaku, Sakai City, Osaka, 591-8025, Japan; b Departments of Pathology, Osaka Rosai Hospital, 1179-3 Nagasone, Kitaku, Sakai City, Osaka, 591-8025, Japan

**Keywords:** Pembrolizumab, PD-L1, Urothelial carcinoma, CR, complete response, CT, computed tomography, MRI, magnetic resonance imaging, TURBT, transurethral resection of the bladder tumor, UC, urothelial carcinoma

## Abstract

A 69-year-old man with a history of non-muscle invasive bladder cancer 12 years ago presented complaining of gross hematuria. He was diagnosed as having invasive T4 bladder cancer with invasion to a branch of the internal iliac artery and received platinum-based chemo-radiation therapy. However, the tumor progressed to extensively infiltrate the pelvic wall, and left leg pain and swelling developed. Pembrolizumab was started, which entirely resolved the tumor after 14 courses of treatment. Pembrolizumab was discontinued after 20 courses of treatment because of adverse events. However, the patient has remained in complete response for over 2 years after pembrolizumab cessation.

## 1 Introduction

Bladder cancer is one of the most common cancers in the world, causing an estimated 17,000 deaths in the United States per year. Platinum-based chemotherapy is used as the first-line treatment for invasive bladder cancer, and the KEYNOTE-045 trial showed the usefulness of pembrolizumab as a second-line treatment.^1^ This trial also reported that the response rate to pembrolizumab was better as PD-L1 expression increased (<1%, 1–10%, and >10%). However, it is still controversial whether the response rate to pembrolizumab is altered by PD-L1 expression in bladder cancer. We report a patient with locally advanced T4 bladder cancer who achieved complete response (CR) with pembrolizumab treatment after progression following chemotherapy and radiation therapy. The patient remains without evidence of recurrence for over 2 years after pembrolizumab therapy was discontinued because of adverse events.

## 2 Case presentation

**Fig. 1:**
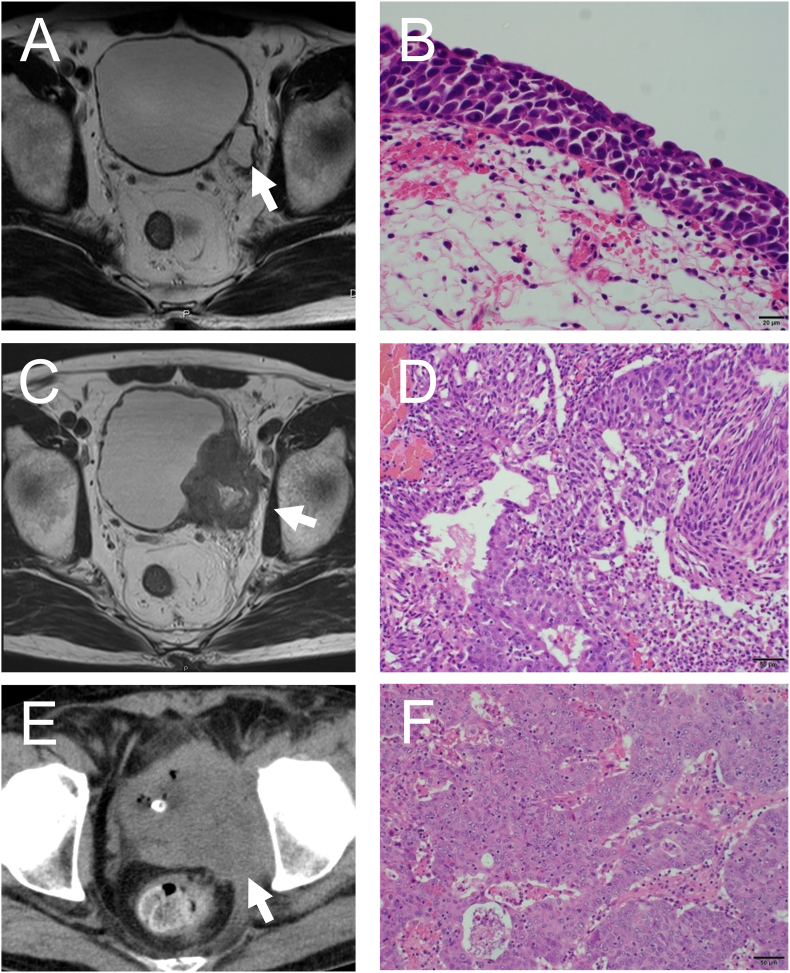
Imaging and pathological findings before and after chemo-radiation. (A) MRI image from 12 years before. The arrow indicates the bladder diverticulum; there was no obvious cancer in the bladder. (B) Histopathological findings of the TURBT 12 years before. Hematoxylin-eosin (HE) staining, × 200. (C) MRI image. The arrow indicates that the cancer was in the bladder diverticulum and had invaded the pelvis and left iliac artery. (D) Histopathological findings of the TURBT before chemo-radiation. HE staining, × 200. (E) CT scan after chemo-radiation. The arrow indicates the progression of disease. (F) Histopathological findings of the TURBT after chemo-radiation. HE staining, × 200.

**Fig. 2:**
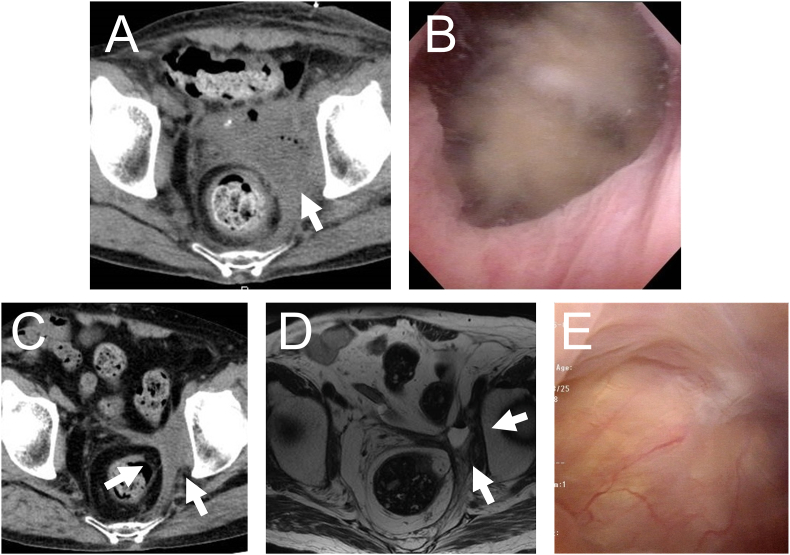
Imaging and cystoscopic findings during treatment with pembrolizumab. (A) CT scan during pembrolizumab treatment. The arrow indicates partial response. (B) Cystoscopy. Necrotic tissue filling the bladder is observed with the cystoscope. (C) CT scan 2 years after discontinuation of treatment. The arrow indicates atrophic bladder and pooling of fluid in the diverticulum. (D) MRI image 2 years after discontinuation of treatment. The arrow indicates atrophic bladder and pooling of fluid in the diverticulum. (E) Cystoscopy 2 years after discontinuation of treatment.

**Fig. 3:**
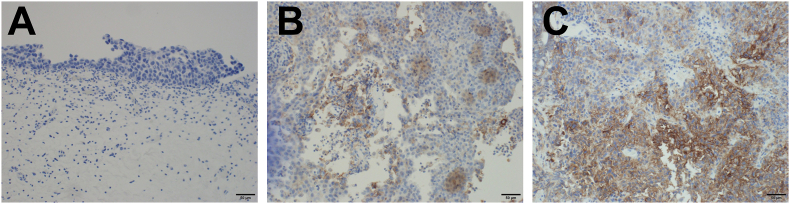
Pathological specimens of previous TURBT immunostained for PD-L1. (A) The first TURBT specimen shows no PD-L1 expression. (B) The pre-chemotherapy TURBT specimen shows 1–5% expression of PD-L1. (C) The post-chemotherapy TURBT specimen shows 10–20% expression of PD-L1.

A 69-year-old Japanese man presented with a complaint of gross hematuria. He had a medical history of non-muscle invasive bladder cancer at 57 years old. Magnetic resonance imaging (MRI) at the time showed a bladder diverticulum but no tumor in the bladder, and the pathological result of transurethral resection of the bladder tumor (TURBT) was carcinoma in situ only in the bladder diverticulum (Fig. 1A and B). After TURBT, he underwent no additional treatment such as intravesical BCG therapy. At this presentation, MRI showed locally invasive T4 bladder cancer with invasion to a branch of the internal iliac artery, which developed from the diverticulum (Fig. 1C). TURBT was performed and pathological examination indicated high-grade invasive urothelial carcinoma (UC) (Fig. 1D). The patient refused total cystectomy and selected bladder preservation therapy. Chemotherapy with cisplatin and gemcitabine plus radiation (60 Gy/30 fr) was performed, but the pathological result of TURBT following treatment was again residual high-grade invasive UC. He underwent second-line chemotherapy combination of methotrexate, vinblastine, epirubicin, and carboplatin, but disease progression continued (Fig. 1E and F), and he developed left leg pain and swelling. Coincidentally, pembrolizumab was approved in Japan just at this time, so pembrolizumab treatment was started. After six courses of treatment, computed tomography (CT) revealed 50% shrinkage of the tumor, which constituted a partial response (Fig. 2A). After nine courses, he experienced repeated urinary retention due to necrotic tissue and require frequent bladder irrigation (Fig. 2B). Due to palliative care of urinary retention, we performed only ureterocutaneous fistula without total cystectomy. After this surgery, the necrotic tissue disappeared. Six courses later, the tumor had completely diminished, and complete remission was determined. After a total of 20 courses treatment, ACTH deficiency and skin rash occurred as immune-related adverse effects of pembrolizumab, and pembrolizumab therapy was discontinued. The patient has remained in complete remission for over 24 months after pembrolizumab cessation (Fig. 2C–E). We retrospectively examined the expression of PD-L1 in each tumor specimen of TURBT at three points with use of the Ventana sp263 assay. Although PD-L1 expression was negative in the initial TURBT, its expression changed to about 5% in the pre-radio-chemotherapy TURBT and to more than 10% after radio-chemotherapy (Fig. 3A–C).

## 3 Discussion

Pembrolizumab was approved by the US Food and Drug Administration in 2017 as a second-line treatment for cisplatin-resistant, locally advanced or metastatic bladder cancer. The mechanism of the drug, which is a humanized monoclonal IgG4 PD-1 antibody, is binding to the PD-1 receptor expressed on T lymphocytes and blocking the signaling of both the PD-L1 and PD-L2 ligands.^2^ Therefore, anti-tumor effect is achieved by disabling the defense mechanism from tumor autoimmunity. The results of the KEYNOTE-045 trial showed a hazard ratio for death of 0.73 for the pembrolizumab group versus the chemotherapy group. However, biomarkers for clinical benefit of immune checkpoint inhibitors are still unclear.

Some reports indicated that PD-L1 expression on tumor cells might be a useful biomarker for immune checkpoint inhibitors.^3^ In the subgroup analysis of the KEYNOTE-045, median OS was prolonged in the patients with higher expression of PD-L1, 8.0 months versus 5.2 months for tumor PD-L1 expression rates >10% versus <10%.^1^ Furthermore, the CR rates of pembrolizumab were 9.1% (10/110) and 7.3% (11/151) for PD-L1 expression of >1% and <1%, respectively.^1^^,^^4^ These results suggested that PD-L1 expression could be a predictor for response of immune checkpoint inhibitors in UC.

Tumor PD-L1 expression was reported to increase after cisplatin-based chemotherapy such as for non-small cell lung cancer,^5^ but there have been no reports in UC. We found dynamic changes of PD-L1 expression in the UC of the present patient as well. Fig. 3 shows that PD-L1 was not expressed in tumor tissues in the initial TURBT but was expressed in 1–5% of TURBT tissue before chemotherapy and in 10–20% after radio-chemotherapy, indicating the effect of therapy. In recent years, avelumab, another immune checkpoint inhibitor, has been approved for maintenance therapy after chemotherapy. Therefore, we need a biomarker to determine whether on-going chemotherapy or immune checkpoint inhibitor maintenance is necessary. Evaluation of PD-L1 expression on tumors after chemotherapy, if possible, might be a useful predictor of response to immune checkpoint inhibitors.

## 4 Conclusion

We report a patient with locally invasive T4 bladder cancer who achieved CR with pembrolizumab following chemo-radiation therapy. The patient has not relapsed for more than 2 years despite the discontinuation of pembrolizumab. We found PD-L1 expression to be dynamically changed by chemotherapy, and the expression rate of PD-L1 just before induction of an immune check inhibitor may be useful as a predictor for treatment response. However, further studies will be needed.

## Sources of funding

This research did not receive any specific grant from funding agencies in the public, commercial, or not-for-profit sectors.

## Declaration of competing interests

None.
